# Enabling Mesenchymal Stromal Cells and Their Extracellular Vesicles Clinical Availability—A Technological and Economical Evaluation

**DOI:** 10.1002/jex2.70037

**Published:** 2025-03-17

**Authors:** Ricardo M. Silva, Sara Sousa Rosa, José A. L. Santos, Ana M. Azevedo, Ana Fernandes‐Platzgummer

**Affiliations:** ^1^ Institute for Bioengineering and Biosciences, Department of Bioengineering Instituto Superior Técnico, Universidade de Lisboa Lisbon Portugal; ^2^ Associate Laboratory i4HB‐Institute for Health and Bioeconomy Instituto Superior Técnico, Universidade de Lisboa Lisbon Portugal

**Keywords:** extracellular vesicles, manufacturing platform, mesenchymal stromal cells, SuperPro Designer

## Abstract

Mesenchymal stromal cell‐derived extracellular vesicles (MSC‐EVs) have shown significant therapeutic potential across a wide range of clinical conditions, complementing the progress of MSC‐based therapies, some of which have already received regulatory approval. However, the high cost of these therapies has limited their accessibility, creating an urgent need to explore manufacturing strategies that reduce the cost of goods and selling prices. This study presents the design and simulation of a scalable manufacturing platform for the co‐production of clinical‐grade MSC and MSC‐EVs using SuperPro Designer. Various production scenarios were evaluated to maximise manufacturing capacity while analysing their impact on economic performance. Our findings demonstrate that for MSC‐EVs doses containing 10^10^ and 10^11^ particles, selling prices range from 166 to 309€ and from 1659 to 3082€, respectively. For clinical doses of MSC, selling prices vary between 965 and 42,673€ depending on dose size and production scale. Importantly, the co‐production approach enables cost‐sharing between products, contributing to significantly lower prices compared to individual production. Overall, the proposed platform achieved an attractive payback time of 3 years and a return on investment of 36%. By increasing the number of staggered production units, further price reductions and improved economic metrics could be attained. In conclusion, this study highlights the potential of the proposed manufacturing platform to deliver cost‐effective, clinical‐grade MSC and MSC‐EVs products, advancing the field of regenerative medicine and enhancing the accessibility of these innovative treatments.

## Graphical abstract

Design and simulation of a manufacturing platform for producing clinical‐grade doses of mesenchymal stromal cells (MSC) and MSC‐derived extracellular vesicles (MSC‐EV). The platform achieves competitive selling prices for MSC doses ranging from 10 to 100 million cells, and offers even more attractive prices for MSC‐EV, with dosages of 10^10^ and 10^11^ EV. Economic analysis indicates the potential for co‐producing MSC and MSC‐EV clinic products, increasing batch output and enhancing competitiveness.

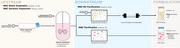

## 1 Introduction

**FIGURE 1:**
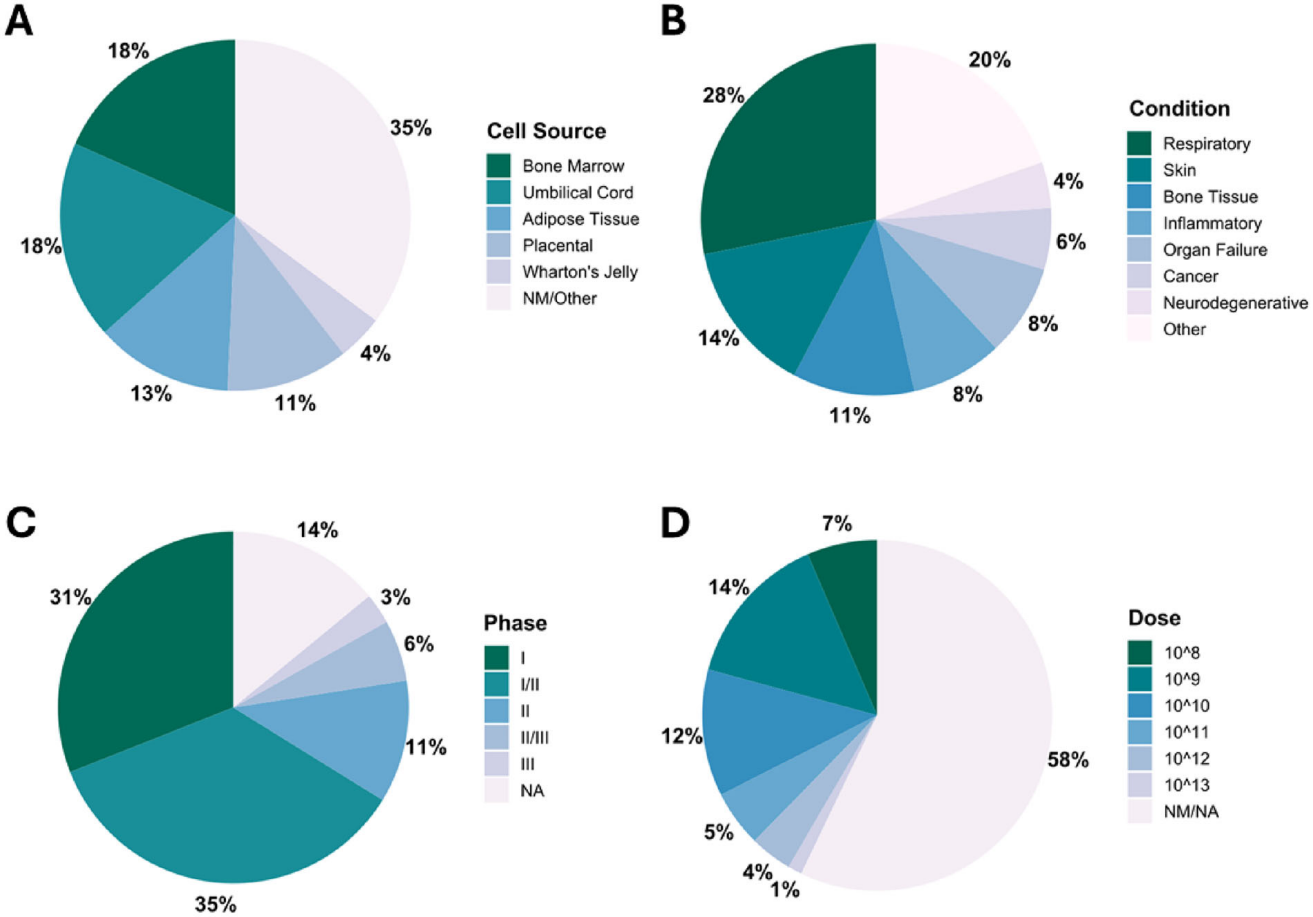
Distribution of clinical trials using MSC‐EVs for therapeutic applications according to MSC Cell Source (A), Condition (B), Phase (C) and EV Dose (D). The information regarding the clinical trials was obtained after a survey in major platforms (https://clinicaltrials.gov/, https://www.isrctn.com/ and https://euclinicaltrials.eu/) using the terms ‘Extracellular Vesicles’, ‘Exosomes’, ‘MSC‐Exosomes’, ‘MSC‐Exos’, ‘MSC‐EVs’. MSC, mesenchymal stromal cells; MSC‐EVs, MSC‐derived extracellular vesicles; NA, not applicable; NM, not mentioned.

Stem cells encompass a diverse range of undifferentiated cells present in the human body that possess the remarkable capacity for self‐renewal and the ability to differentiate into multiple cell lineages (Zakrzewski et al. 2019; Ullah, Subbarao, and Rho 2015). Because of their unique properties, these cells have been subjected to extensive research, particularly in the fields of regenerative medicine and tissue engineering. Among the various types of stem cells, mesenchymal stromal cells (MSC) present distinct characteristics that position them as leading candidates for cell‐based therapies. Not only they are widely available (Soure et al. 2016; Kim and Park 2017; Rastegar et al. 2010), with high proliferative potential in vitro (Kim and Park 2017; Rastegar et al. 2010; Prodinger et al. 2017), but studies have also highlighted their reduced ethical and safety concerns (Zakrzewski et al. 2019; Kim and Park 2017), their capacity to migrate to inflamed or damaged tissues following administration (Rastegar et al. 2010; Zhou et al. 2021), and the reduced risk of adverse immune response (Kim and Park 2017; Zhou et al. 2021; Ankrum, Ong, and Karp 2014). The therapeutic potential of MSC was initially attributed to their homing potential, engraftment capacity in damaged tissue and self‐healing stimulation (Mendt, Rezvani, and Shpall 2019; Buzhor et al. 2014). However, it is now understood that their therapeutic effects also stem from their microenvironment and the bioactive molecules they secrete (Kordelas et al. 2014). In addition to soluble factors, growth factors and chemokines, the MSC ‘secretome’ is rich in extracellular vesicles (EVs), including microvesicles (MVs; 100–1000 nm) and exosomes (30–150 nm) (Mendt, Rezvani, and Shpall 2019; Caplan and Dennis 2006; Baek et al. 2019). As valuable information carriers, MSC‐derived EVs (MSC‐EVs) have emerged as promising therapeutic vehicles, often demonstrating comparable or enhanced efficacy compared to parental MSC (Zhou et al. 2021; Kordelas et al. 2014; Bian et al. 2019; Bruno et al. 2009). EVs offer distinct advantages as a cell‐free therapy, including biocompatibility, non‐self‐replication (Kordelas et al. 2014) and non‐immunogenicity (Baek et al. 2019). Moreover, EVs exhibit a remarkable ability to deliver cargo and cross the blood‐brain barrier (Corbett et al. 2019), surpassing the common limitations faced by many conventional drugs. Unlike MSC, EVs also avoid entrapment in capillaries following intravenous administration (Baek et al. 2019). MSC have demonstrated therapeutic effectiveness in a wide range of conditions, including Parkinson's (Inna et al. 2007), Alzheimer's disease (Danielyan et al. 2014), graft‐versus‐host disease (GvHD) (Le Blanc et al. 2008; Ringden and Le Blanc 2011), Chron's disease (Rachelle et al. 2015; Carvello et al. 2019), cardiovascular conditions (Noritoshi et al. 2005, Hare et al. 2009), liver injuries (Kharaziha et al. 2009, Lu et al. 2018) and many others (Naghdi et al. 2009; Henryk et al. 2001). These compelling findings have encouraged further research and investment (Reisman and Adams 2014). Over the past decade, the number of clinical trials involving MSC has steadily increase (Ullah, Subbarao, and Rho 2015, Kabat et al. 2020; Jovic et al. 2022; Childs et al. 2020), but only eleven MSC‐based advanced therapy medicinal products (ATMPs) have received market approval (Table 1) (Childs et al. 2020; Pereira Chilima, Moncaubeig, and Farid 2018; Ramezankhani et al. 2020, Johnson et al. 2021; Alliance for Regenerative Medicine; Wang et al. 2021; Wright, Arthaud‐Day, and Weiss 2021). The list, however, gets larger considering MSC‐differentiated cell lines (e.g., chondrocytes, osteoblasts) (Childs et al. 2020; Alliance for Regenerative Medicine; Najar et al. 2021). Prochymal (Remestemcel‐L) stands out as the world's first approved stem cell‐based ATMP for the treatment of acute GvHD in children, following regulatory approval by Canada (Rattue) and New Zealand (Waltz 2013) agencies. In Europe, the EMA has only approved one MSC therapy, Alofisel for treating complex anal fistulas in adult patients with Crohn's disease (Alofisel | European Medicines Agency). In the United States, where the most MSC clinical trials are conducted, besides Remestemcel‐L, only a limited number of ATMPs involving hematopoietic progenitor cells have received Food and Drug Administration (FDA) approval (U. S. Food and Drug Administration). EVs research has shown efficacy in treating a wide‐range of conditions including ischemic diseases (Bian et al. 2019), chronic kidney disease (Nassar et al. 2016), graft‐versus‐host disease (Kordelas et al. 2014), lung diseases (Fujita et al. 2018) and others (Reza‐Zaldivar et al. 2019; Börger et al. 2017). Although no MSC‐EVs or EV‐based therapy has received regulatory approval to date, the number of registered clinical trials have been rising. In 2022, Lofty and colleagues identified 14 ongoing clinical trials using MSC‐EVs (Lotfy, AboQuella, and Wang 2023). By 2024, this number has surpassed 70, covering a diverse array of conditions and development stages (Figure 1). While some commercial MSC‐EVs products are already available, they have not undergone rigorous regulatory evaluation. The Centers for Disease Control and Prevention (CDC) have issued cautionary statements highlighting the potential risks associated with unregulated MSC‐EV products (Stem Cell and Exosome Products | HAI | CDC 2019).

**TABLE 1:** MSC‐based ATMPs with marketing authorisation.

Product/Year Company	Clinical indication	Dose size	Price/dose (€) (complete treatment)
Queencell/2010 Anterogen Co, Ltd.	Subcutaneous tissue defect	$70\times 10^{6}$ cells (Childs et al. 2020)	N.A.
Cellgram‐AMI/2011 Pharmicell Co, Ltd.	Acute myocardial infarction	$50-90\times 10^{6}$ cells (Childs et al. 2020; Ramezankhani et al. 2020)	∼13,500 (Childs et al. 2020; Ramezankhani et al. 2020)
Cartistem/2012 Medipost Co, Ltd.	Osteoarthritis	∼$10\times 10^{6}$ cells (Medipost)	∼17,100–19,000 (additional 9000 for extra treatment) (Childs et al. 2020; Ramezankhani et al. 2020)
Cupistem/2012 Anterogen Co, Ltd.	Chron's fistula	$30-60\times 10^{6}$ cells (Ramezankhani et al. 2020)	∼2700–4500 (Ramezankhani et al. 2020)
Prochymal/2012 Mesoblast Ltd.	Graft versus Host Disease	$100\times 10^{6}$ cells (8–12 treatments) (Childs et al. 2020; Mesoblast 2014; Mack 2009; Galipeau and Sensébé 2018)	∼18,000 (∼180,000) (Childs et al. 2020; Pereira Chilima, Moncaubeig, and Farid 2018; Ramezankhani et al. 2020)
Neuronata‐R/2014 Corestem Inc.	Amyotrophic lateral sclerosis	$40\times 10^{6}$ cells (Corestem) (24 treatments)	∼2062 (49,500) (Ramezankhani et al. 2020)
Temcell HS/2015 JCR Pharmaceuticals	Graft versus Host Disease	$72\times 10^{6}$ cells (12–24 treatments) (Childs et al. 2020; Ramezankhani et al. 2020; Galipeau and Sensébé 2018)	∼6370 (153,000) (Galipeau and Sensébé 2018)
Stempeucel/2016 Stempeutics R.	Buerger's disease	$1-2\times 10^{6}$ cells/kg (Ramezankhani et al. 2020)	∼1980 (Ramezankhani et al. 2020)
Alofisel/2018 Takeda	Crohn's perianal fistula	$120\times 10^{6}$ cells (National Institute for Health and Care Excellence; Takeda Pharma)	∼64,000 (National Institute for Health and Care Excellence)
Stemirac/2018 Nipro Corp.	Spinal cord injury	$50-200\times 10^{6}$ cells (Nipro Corporation)	121,500 (Childs et al. 2020)
MesestroCell/2019 CellTech Pharmed	Osteoarthritis	$2-4\times 10^{7}$ cells (Ramezankhani et al. 2020)	N.A.

*Note*: This list presents approved products containing MSC, providing information on disease target, dose sizes, selling prices per dose and complete treatment prices if multiple doses are required. The price was calculated in euros (€) according to the exchange rate of 1 USD = 0.90€ at 24th of July of the year 2023.

Although MSC and MSC‐EVs have demonstrated significant potential in preclinical studies and early‐stage clinical trials, numerous clinical trials have faced challenges, including limited efficacy in specific conditions and adverse immune responses. These challenges highlight the need for more refined therapeutic strategies. To meet the rigorous standards required for clinical applications, several critical issues must be addressed, particularly in their manufacturing process. This includes achieving technological scalability and ensuring process efficiency. Additionally, high capital investment and elevated cost of goods (COG) present substantial barriers to the widespread adoption of MSC and EVs therapies. Addressing these challenges necessitates a comprehensive evaluation of their economic viability to ensure these therapies can achieve both success and accessibility for a broader patient population. In this article, we present a model developed using SuperPro Designer to simulate the large‐scale co‐production and co‐purification of pharmaceutical‐grade MSC and MSC‐EVs, covering all steps from MSC thawing to product formulation. By combining information from existing literature with advances from our research group (Fernandes‐Platzgummer et al. 2023; Silva et al. 2023), we provide a comprehensive process overview. Furthermore, leveraging the software's integrated tools, we evaluated the operational and economic performance of the manufacturing process, enabling an assessment of its profitability. This proposed manufacturing platform demonstrates the ability to deliver therapeutic doses of both MSC and MSC‐EVs at competitive selling prices, highlighting the cost‐effectiveness of the MSC‐EVs approach.

Although simulation‐based studies cannot fully predict real‐world outcomes, they provide valuable insights for optimising production protocols and reducing costs, thereby paving the way for future experimental validation. Moreover, while MSC are currently in advanced clinical development stages, EVs are emerging as a promising therapeutic platform, with early‐stage trials showing potential in tissue regeneration and immune modulation. This work, therefore, represents a foundational step toward optimising production methods, reducing costs and enhancing the scalability of both MSC and MSC‐EVs therapies.

## 2 Materials and Methods

### 2.1 Biomanufacturing Considerations

This section outlines the assumptions made in the simulation of the manufacturing process for both upstream and downstream of EVs production using MSC cells. To enhance the sustainability of the process, we have added a sector where MSC cells are also processed to be marked as a by‐product. The detailed process is described below.

### 2.2 Upstream Processing

The scalability and culture conditions of manufacturing processes are critical when producing large quantities of MSC for clinical application. While different cultivation parameters can be used to maximise EVs production, such changes can also negatively impact the biological functionality of both the cells and their secreted EVs (Takeda Pharma; Nipro Corporation). To maximise production, 3‐dimensional (3D) culture platforms have emerged as a preferred option, as they have been shown to yield a 20‐fold increase in EV production compared to traditional 2D approaches (Haraszti et al. 2018) while simultaneously reducing manufacturing costs (Adlerz et al., 2020; Pereira Chilima, Moncaubeig, and Farid 2018).

#### 2.2.1 MSC Thawing

Although the MSC manufacturing process typically starts with MSC isolation from donor tissues, in our simulation, we have chosen to use cryopreserved MSC from cell banks. Although the impact of cryopreservation on cell function after thawing, is not universally agreed upon, it remains crucial for the transportation of final doses and for cell banking (Bahsoun, Coopman, and Akam 2020; Bahsoun, Coopman, and Akam 2019). Following thawing, cells usually undergo centrifugation to remove cryoprotectants, such as dimethyl sulfoxide (DMSO). However, centrifugation is labour‐intensive and sometimes a single centrifugation step may not be sufficient, especially on a large scale. In this case, a less labour‐intensive method, such as microfiltration, can be employed to ensure high efficiency and scalability in DMSO removal (Hornberger et al. 2019).

#### 2.2.2 Adherent Cell Culture

MSC are anchorage‐dependent cells, meaning they require a suitable surface for ex vivo expansion. In 2D cultures, such as T‐Flasks or Cell Factories, there is typically sufficient surface area for cell adhesion. However, when aiming for large scale manufacturing, this strategy is often associated with lower production yields (Haraszti et al. 2018). Alternatively, 3D culture systems, such as stirred bioreactors, offer improved process control and scalability (Adlerz et al., 2020; Soure et al. 2016; Dos Santos et al. 2014), especially when combined with microcarriers for cell attachment (Soure et al. 2016). Microcarriers, which are low‐density solid or porous spheres with diameters ranging between 100 and 300 µm, offer a higher surface‐to‐volume ratio, enabling the cultivation of a high number of cells per unit of volume (Tsai and Ma 2016; Koh et al. 2020; Hu 2012). Typically, microcarriers are made of materials such as glass, gelatine, dextran, polystyrene, amongst others (Koh et al. 2020; Hu 2012). Among the many options, polystyrene is often chosen due to its cost‐effectiveness and ease of use. It can be easily sterilised and MSC exhibit strong adherence to plastic substrates in culture media containing serum or its derivatives, facilitating cell attachment. However, transitioning to serum‐free culture media, which is preferred to avoid complications associated with serum and its derivatives, may pose challenges in achieving cell attachment. To address this, coating substrates can be employed to enhance cell adhesion (Hornberger et al. 2019) and promote consistent growth, reducing batch variability and improving overall system efficacy (Ryan n.d.).

#### 2.2.3 MSC Growth and MSC‐EVs Production

The growth of adherent cells is typically divided into three different stages: an initial lag phase, mainly associated with the adhesion period lasting 1–2 days; followed by an exponential phase, corresponding to cells’ exponential growth that occurs for 4–6 days; and finally, a stationary phase, in which the proliferation rate decreases (Somal et al. 2016). For anchorage‐dependant cells, like MSC, the stationary phase is typically reached when cells have reached maximum confluence due to limited available surface area or due to nutrient depletion and/or accumulation of inhibitory metabolites, such as lactate and ammonia. At this point, the number of viable cells begins to decline, eventually leading to cell death (Hu 2012). However, none of these stages can be achieved without proper medium supplementation, as this plays a critical role on cell growth. Essential components include glucose, amino acids, vitamins, nucleotides, salts, growth factors and others. Glucose and glutamine are particularly critical as energy sources and as carbon and nitrogen sources, respectively. Concomitantly, cell generate and release by‐products including carbon dioxide, water, lactate and ammonia (Hu 2012).

### 2.3 Downstream Processing

The downstream processing involves the separation and purification of EVs from MSC, debris, proteins, and other impurities present. Purifying EVs is significantly more challenging than their MSC counterparts, with various techniques exploring the physical‐chemical and biological properties of EVs. Currently, ultracentrifugation is the most commonly used method for EVs purification. However, this method is difficult to scale and frequently yields impure final products unsuitable for clinical use (Baranyai et al. 2015; Doyle and Wang 2019; Nordin et al. 2015). Alternative methods, such as ultrafiltration, precipitation, size exclusion chromatography and immunoaffinity‐based methods have been proposed, but each has associated disadvantages, including lack of standardisation or drawbacks in terms of purity, recovery or scalability. On the other hand, ion‐exchange chromatography is a well‐established and scalable method widely used for the large‐scale manufacturing of proteins and larger biomolecules (Steppert et al. 2016; Moleirinho et al. 2018).

Building on our previous work, we developed a chromatography‐based platform to achieve highly purified EVs in large quantities. In this context, various anion‐exchange alternatives were evaluated for EVs purification. Capto Q ImpRes proved to be the most effective resin, and the integrated platform allows the recovery of more than 50% of EVs with purity standards in accordance to regulatory agencies (Silva et al. 2023). These findings were replicated in the simulation process design, which included the pre‐purification step, nuclease treatment, and finally, anion‐exchange chromatography using Capto Q ImpRes. To calculate the resin binding capacity for EVs, 100 mL of pre‐treated conditioned medium containing $5.85\times 10^{12}$ EVs (0.026 g) was loaded into a 1 mL column. No EVs were detected in the flow through of collected fractions, suggesting that the resin binding capacity for EVs had not been reached. A resin binding capacity 0.26 mg EV/mL of resin was then considered.

### 2.4 Dosage Considerations

**FIGURE 2:**
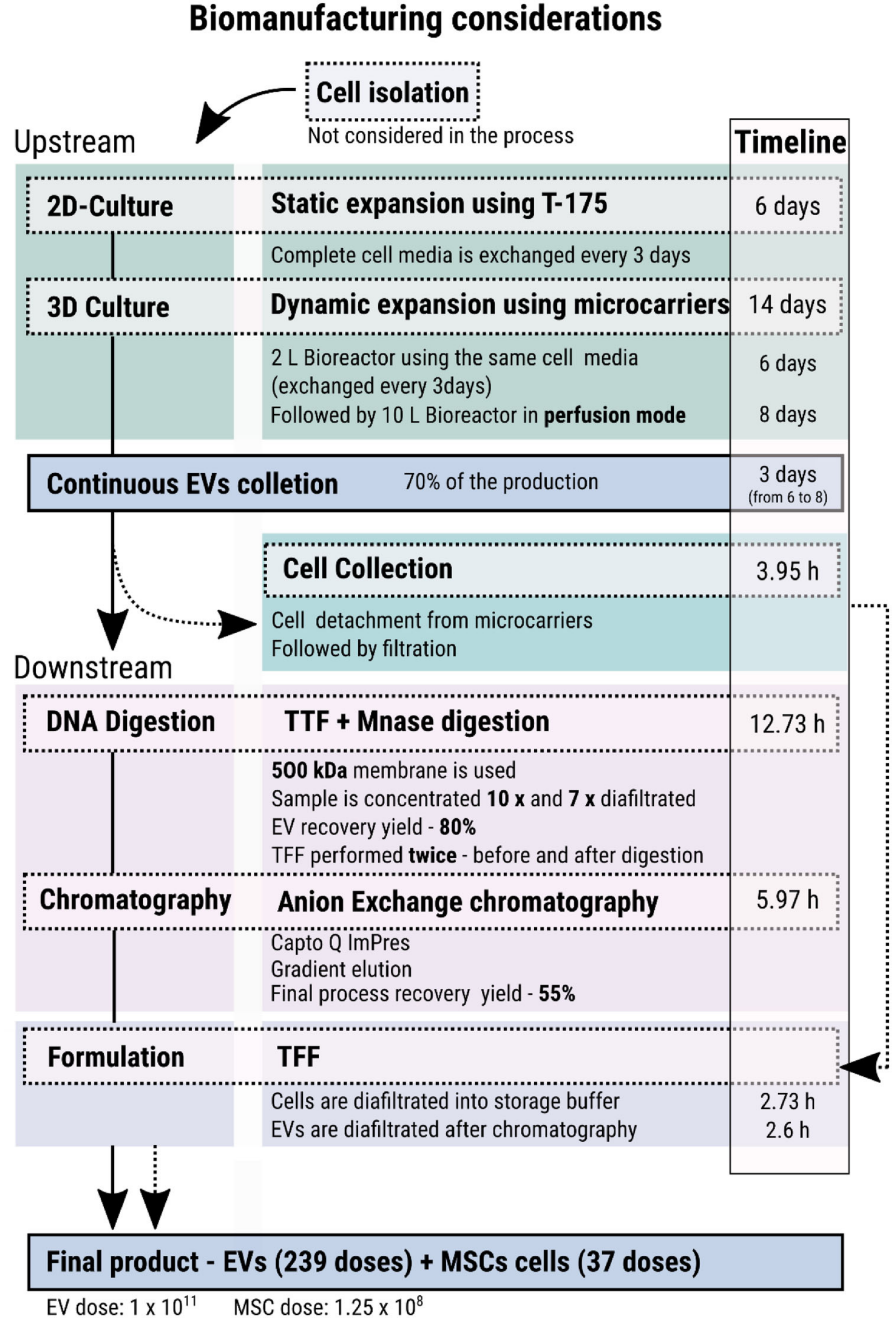
Diagram of the process for the production of 239 doses of EVs and 37 doses of MSC cells with the method described in Section 2. EVs, extracellular vesicles; MSC, mesenchymal stromal cells; TFF, tangential flow filtration.

Reported doses sizes for MSC range from 10^6^ to 10^9^ cells per patient (Kabat et al. 2020; Pereira Chilima, Moncaubeig, and Farid 2018; Parekkadan and Milwid 2010; Simaria et al. 2014; Homma et al. 2023), while for MSC‐EVs, the recommended dose size varies from 10^7^ to 10^13^ particles per dose (Mendt, Rezvani, and Shpall 2019; Lotfy, AboQuella, and Wang 2023; Homma et al. 2023, Moleirinho et al. 2019; Maumus et al. 2020; Phinney and Pittenger 2017) (Figure 1). In this work, a dosage of $1\times 10^{11}$ EVs and $1.25\times 10^{8}$ MSC are considered (Figure 2).

### 2.5 Process Description

**FIGURE 3:**
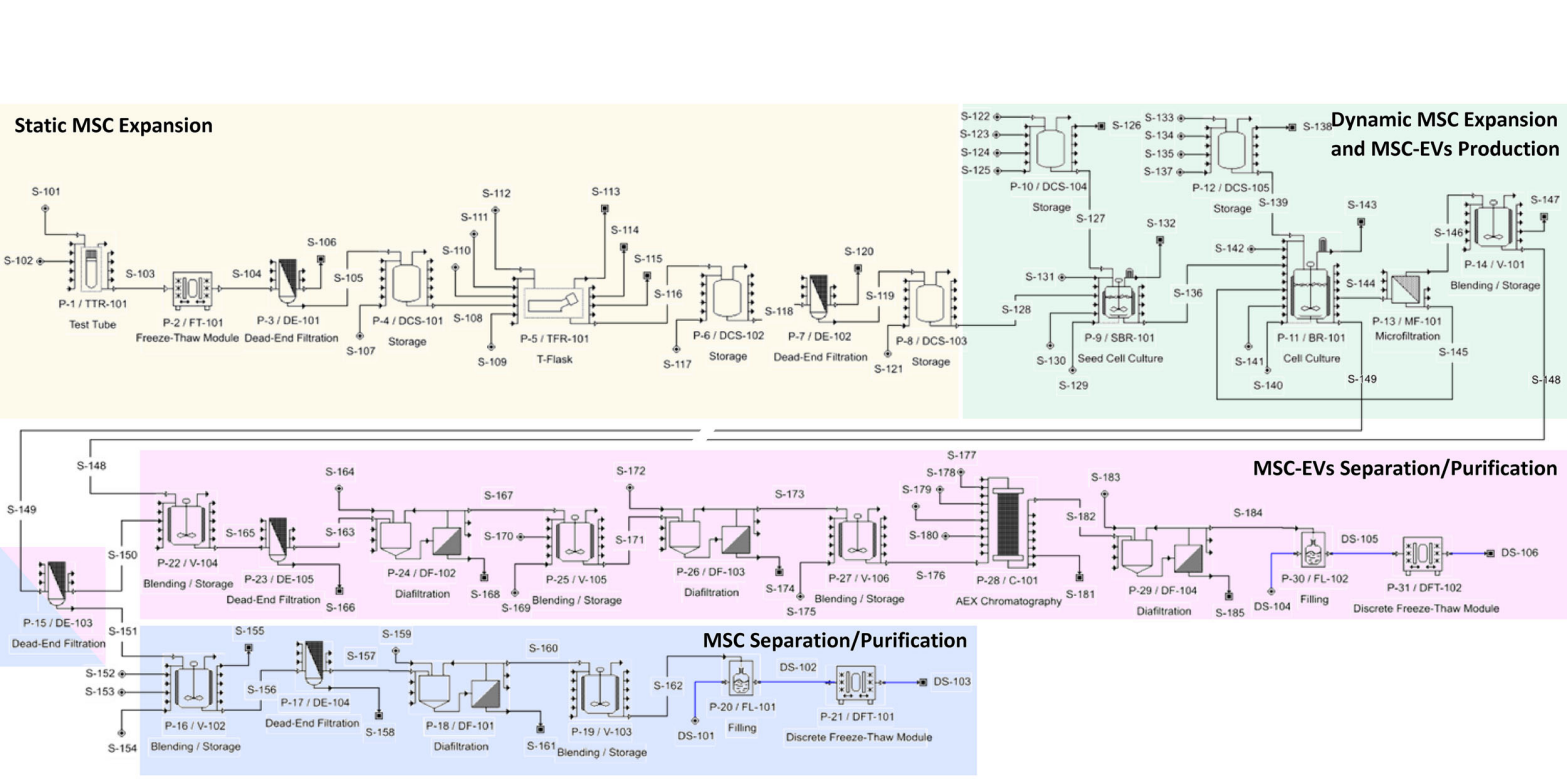
Schematic workflow representation of the MSC and MSC‐EVs production line. The four sections that comprise this project are identified in different colours. Yellow: Static MSC expansion; Green: Dynamic MSC culture and MSC‐EVs production; Pink: MSC‐EVs separation/purification; Blue: MSC separation/purification. The dead‐end filtration unit DE‐103 separates MSC from MSC‐EVs and is identified as a member of separate sections. AEX, anion exchange; MSC, mesenchymal stromal cells; MSC‐EVs, MSC‐derived extracellular vesicles.

The EVs manufacturing process was simulated using SuperPro Designer (Intelligen Inc., Scotch Plains, NJ, USA; version 10). The model combines published data with findings from our research group (Silva et al. 2023; Dos Santos et al. 2014; Fernandes‐Platzgummer et al. 2016), as previously described. To enhance the utility of the process, co‐production of MSC‐EVs and MSC was explored, leveraging the same manufacturing platform. The proposed manufacturing platform consists of an upstream section dedicated to MSC expansion and MSC‐EVs production, with two main sectors identified: a static 2D culture followed by dynamic MSC expansion in bioreactors with capacities of 2 and 10 L. Subsequently, the downstream section comprises two sectors, one for the separation and purification of MSC and the other for MSC‐EVs. A diagram summarising key process details is presented in Figure 2, while the complete process flowchart is illustrated in Figure 3. In the latter it is possible to identify the four main process sectors, each assigned a different colour on the flowsheet: (i) static expansion (yellow), (ii) dynamic MSC culture and MSC‐EVs production (green), (iii) separation/purification of MSC (blue) and (iv) separation/purification of EVs (pink).

### 2.6 Upstream Processing

The upstream section is divided in two sectors: static expansion and dynamic MSC culture.

#### 2.6.1 Static Expansion

The process begins with thawing 1 mL cryogenic vials (TTR‐101) containing the ‘seed’ MSC in a cryogenic solution at 37°C using thawing medium (FT‐101) at a dilution of 1:4, containing Dulbecco's ’Modified ’Eagle Medium (DMEM) composed of glucose, inorganic salts, amino acids, vitamins and other components, along with 20% fetal bovine serum (FBS) (Fernandes‐Platzgummer et al. 2016). After thawing, cells are recovered by dead‐end filtration (DE‐102), using a 0.45 µm membrane, which retains MSC (10–20 µm) and removes smaller contaminants (Hu 2012; Lodish et al. 2000). The retained cells are resuspended in 1 mL of expansion medium and plated in T‐175 flasks (TFR‐101). The expansion medium composition follows the guidelines presented in Fernandes‐Platzgummer et al. (2016), which includes amino acids (1%) and 97.9% of complete medium, composed of a carbon source (99%) and inorganic salts, vitamins (1%). Cells are seeded at $3\times 10^{3}$ cells/cm*^2^* and cultured to 80% confluency, typically achieved within 6 days. On Day 3, the culture medium is refreshed by removing the exhausted medium and adding 20 mL of fresh expansion medium. On Day 6, cells are washed with 80 mL of phosphate‐buffered saline (PBS) solution per T‐175 flask, followed by the addition of 7 mL of protease solution. At the end, a total of $4.85\times 10^{6}$ cells are harvested per T‐175 flask. To inoculate the seed bioreactor with $5\times 10^{7}$ cells, 11 T‐175 flasks are used, requiring 11 cryogenic vials. At the end of the static expansion phase, cells are resuspended in 500 mL of expansion medium and filtered using a 0.2 µm membrane (DE‐103) for sterility.

#### 2.6.2 Dynamic Expansion

The cells harvested from the T‐flasks are transferred into the 2 L Seed Bioreactor (SBR‐101) containing 20 g of cross‐linked polystyrene plastic microcarriers, with a surface area of 360 cm^2^/g, and 200 mL of fresh expansion culture medium. The microcarriers are pre‐coated (DC‐104) with 11 µL/cm^2^ of coating solution. Inoculation is performed with 600 mL of cell suspension at a seeding density of $8.71\times 10^{4}$ cells/mL. The cells adhere to the microcarriers within 24 h, assuming a 100% yield. From Day 3 to 6, the remaining 1.4 L of expansion medium is added, and by the end of day 6, $1\times 10^{9}$ cells are theoretically reached. $9.97\times 10^{8}$ cells are harvested from SBR‐101 and transferred to a 10 L Bioreactor (BR‐101), which contains 4 L of fresh expansion culture medium and freshly coated microcarriers from DCS‐105. This expansion cycle takes 8 days, with the bioreactor operating under perfusion mode from Day 4. The fresh culture medium is continuously added at a constant flow rate of 59 mL/h, while the exhausted medium is filtered through MF‐101 (0.45 µm) to remove metabolites. Invariably, some products of interest may also pass through the 0.45 µm membrane. To prevent EVs loss, the 6.5 L permeate stream is directed to a 10 L collection tank (V‐101). However, since in most protocols, EVs collection typically initiates at later stages of cellular growth (Gaesser et al. 2024; de Almeida Fuzeta et al. 2020; Adlerz et al. 2019), the collection volume between Days 4 and 6—4.4 L—is discarded, while the remaining 2.6 L collected after Day 6 is reintroduced back into the downstream section for the separation/purification of EVs.

### 2.7 Macrochemical Equations

The metabolism of the cell culture process can be described by a stoichiometric equation governing both stages. Glucose, amino acids and oxygen are the key elements of the equation, along with unspecified but essential macro‐/micro‐nutrients such as vitamins, lipids and nucleotides. The main products of the process are MSC and EVs, representing cells and their secreted microvesicles, respectively. Metabolites, particularly ammonia and lactate, are identified as by‐products. Therefore, the overall production of MSC and MSC‐EVs can be represented by Equation (1), where the lowercase letters represent the mass stoichiometric coefficients.

(1) $$\begin{array}{ccc} a\mathrm{Glucose} & + & b\mathrm{Amino}\;\mathrm{acids}+c\left(\mathrm{Macro}-/\mathrm{Micro}-\mathrm{nutrients}\right)\\ & + & dO_{2}\to \mathrm{eMSC}+\mathrm{fEV}+\mathrm{gC}O_{2}+hH_{2}O+i\mathrm{Metabolites} \end{array}$$

MSC secreted EVs can be further divided into two categories based on their size, namely, exosomes (EV1), which are smaller, ranging from 30 to 150 nm, and microvesicles (EV2), which can reach up to 5000 nm if apoptotic bodies are considered. The distribution of EV according to their size in MSC secretome is not clearly described but seems to be dependent on medium composition. Figueroa‐Valdés et al. described exosomes fluctuating between 50% and 70% of the total particles (Figueroa‐Valdés et al. 2021). In the current simulation, the mean value was assumed, with 60% of the secreted EV being EV1 and 40% being EV2. Equation (2) represents the division of the secreted EVs to the respective categories of exosomes (EV1) and remaining vesicles (EV2).

(2) 100EV→60EV1+40EV2

Both Equations (1) and (2) operate in parallel in both 2D and 3D culture systems during their respective expansion times. To accurately represent cell expansion, some parameters require clarification. First, the mass of cells and vesicles needed to be defined. The available literature reports mammalian cells with a diameter of 12–18 µm (Hu 2012), but to simplify calculations a cube‐shaped cell with a 15 µm edge length is considered. A cell mass of $3.5\times 10^{-9}$ *g* has been proposed (Lodish et al. 2000). For EVs, a generic spherical shape with a diameter of 200 nm and a density of $1.08\;g/cm^{3}$ was assumed (Brennan et al. 2020; Liangsupree, Multia, and Riekkola 2021). With these values, the mass of a single EVs was calculated to be $4.52\times 10^{-15}$ g. This value is one order of magnitude higher than the estimation of Sverdlov (Sverdlov 2012) but two orders of magnitude lower than that of Stratton et al. (2014). These differences can be attributed to the heterogeneity in the size of the EVs population. Additionally, the oxygen consumption rate of MSC is reported to be in the range of 30–65 fmol/h/cell (Krinner et al. 2009; Lavrentieva et al. 2010), with a consumption rate of 47.5 fmol/h/cell assumed. Determining successful stoichiometric coefficients also relies on establishing consumption ratios between reagents and products as depicted in the cell growth equation. The most relevant ratios include the number of cells formed per volume of expansion culture medium and the number of EVs secreted by each cell.

In this design, 2D culture systems can generate up to $3.6\times 10^{5}$ cells/mL (Adlerz et al. 2019; Hassan et al. 2015), while 3D expansion systems, particularly microcarrier‐based bioreactors, report results ranging from $1.5-6.7\times 10^{5}$ cells/mL (Dos Santos et al. 2014; de Almeida Fuzeta et al. 2020; Adlerz et al. 2019; Lawson et al. 2017). Although the latter being commonly praised with higher cell outputs, these values should be carefully interpreted due to the various factors that can influence cell yield, such as culturing conditions and strategies employed (Lawson et al. 2017). For calculations in the current simulation, a constant cell concentration of $5\times 10^{5}$ cells/mL was considered for all expansion units. Particle‐cell ratio is challenging to define, and the information available is limited. Studies in this field report particle‐cell ratios as low as $5\times 10^{2}$ particles/cell (Kordelas et al. 2014, Phelps et al. 2022) and as high as $1\times 10^{5}$ particles/cell (Haraszti et al. 2018), with most values falling between—$10^{3}$ particles/cell and $10^{4}$ particles/cell (Mendt, Rezvani, and Shpall 2019; de Almeida Fuzeta et al. 2020; Adlerz et al. 2019; Figueroa‐Valdés et al. 2021, Phelps et al. 2022; Kim et al. 2020). However, it is important to note that the calculated ratio may not accurately reflect reality since, in many cases, this value is estimated based on a series of downstream steps that typically reduce the total number of particles. For instance, Haraszti et al. calculate different particle‐cell ratios depending on the isolation step (Haraszti et al. 2018). Considering these factors, a conservative estimate of $1.5\times 10^{4}$ particles/cell was considered. With the production ratios established, the mass stoichiometric equations governing MSC expansion and EVs production can be established for each cell culture step. For the 2D culture system, TFR‐101, Equation (3) describes the first 72 h, while Equation (4) describes the following 72 h. For the 3D expansion unit, SBR‐101, Equation (4) describes the entire 144‐h expansion process. For the 10 L Bioreactor, BR‐101, Equation (4) describes the first 72 h, while Equation (5) describes the final 120 h. The only limitation is, as aforementioned, cell growth is not constant throughout time.

(3) $$\begin{array}{ccc} 100G\mathrm{lucose} & + & 20.63\mathrm{Amino}\;\mathrm{acids}\\ & + & 1.01\left(\mathrm{Macro}-/\mathrm{Micro}-\mathrm{nutrients}\right)+0.56O_{2}\\ & \to & 18.06\mathrm{MSC}+0.48\mathrm{EV}+0.79CO_{2}+51.44H_{2}O\\ & + & 51.44\mathrm{Metabolites} \end{array}$$

(4) $$\begin{array}{ccc} 100\mathrm{Glucose} & + & 20.63\mathrm{Amino}\;\mathrm{acids}\\ & + & 1.01\left(\mathrm{Macro}-/\mathrm{Micro}-\mathrm{nutrients}\right)+0.56O_{2}\\ & \to & 18.06\mathrm{MSC}+0.35\mathrm{EV}+0.79CO_{2}+51.51H_{2}O\\ & + & 51.51\mathrm{Metabolites} \end{array}$$

(5) $$\begin{array}{ccc} 100\mathrm{Glucose} & + & 20.63\mathrm{Amino}\;\mathrm{acids}\\ & + & 1.01\left(\mathrm{Macro}-/\mathrm{Micro}-\mathrm{nutrients}\right)+0.94O_{2}\\ & \to & 18.06\mathrm{MSC}+0.35\mathrm{EV}+1.32CO_{2}+51.43H_{2}O\\ & + & 51.43\mathrm{Metabolites} \end{array}$$

The rationale behind calculating each stoichiometric coefficient is similar for all equations. The stoichiometric coefficient for the carbon/energy source, glucose, was fixed at 100. Furthermore, based on in‐house results and available literature (Fernandes‐Platzgummer et al. 2016), we know the number of seeding cells as well as the target number of cells to be achieved at the end of each culturing step (Table 2). For the first 72 h of TFR‐101, we assumed that 40% of the final number of cells have already been produced, while for the same period in the 3D expansion units—SBR‐101 and BR‐101—only 20% has been produced. Knowing the final cell concentration, we can easily calculate the required quantity of expansion culture medium needed to generate it. Considering that glutamine can fulfill energy needs and still and serve as building blocks for protein formation, we calculate a stoichiometric coefficient of 20.63 for glutamine, making it the second most consumed reagent in MSC expansion. This value is supported by early findings, as Hu et al. reported that glutamine consumption rate lies between 10% and 20% of glucose consumption (Hu 2012). Other nutrients are usually not included in this type of equation due to their typically low consumption. In the current equation, these macro‐/micro‐nutrients correspond to 1%. Comparing the MSC expansion equations, we can observe a few differences. As aforementioned, Equation (5) governs the 120‐h period of BR‐101 instead of the previously considered 72 h, which explains the variations in the oxygen and carbon dioxide stoichiometric coefficients. For Equations (3) and (4), the disparities lie precisely in the stoichiometric coefficient of EVs. Equation (3) illustrates the process during the first 72 h of the 2D expansion unit, while Equation (4) describes the operation throughout the 3D expansion. In other words, the EVs coefficient in Equation (3) corresponds to those secreted by the seeding cells (thawed cells) plus the cells formed during cell growth, whereas Equation (4), like Equation (5), only considers the cells formed during the specific period described by the equation.

**TABLE 2:** Key process parameters, MSC and EVs values, considered throughout the designed process.

Process parameter	Value
Seeding density (TFR‐101)	3000 cells/cm^2^
Harvest (TFR‐101)	∼$5.33\times 10^{7}$ cells
Seeding (SBR‐101)	∼$5.23\times 10^{7}$ cells
Harvest (SBR‐101)/Seeding (BR‐101)	∼9.9$\times 10^{8}$ cells
Cell lysis (BR‐101)	10%
Harvest (BR‐101)	∼5.4$\times 10^{9}$ cells
Number of MSC (S‐162)	∼4.73$\times 10^{9}$ cells
Number of EVs (S‐184)	∼2.39$\times 10^{13}$ particles
Volume (SBR‐101)	2 L
Volume (BR‐101)	10 L

Abbreviations: EVs, extracellular vesicles; MSC, mesenchymal stromal cells.

Following the static expansion, cells continue to expand in a dynamic culture system, specifically the 2 L Seed Bioreactor, SBR‐101. Published literature on MSC reports seeding concentrations ranging from $1.5\times 10^{4}$ to $1\times 10^{5}$ cells/mL (Fernandes‐Platzgummer et al. 2016; Lawson et al. 2017; Tsai and Pacak 2021). We consider inoculate the SBR‐101 with $5\times 10^{7}$ cells at an initial concentration of $8.3\times 10^{4}$ cells/mL. To achieve this, at least 11 TFR‐101 units operating in parallel are required (a single static unit produces $4.85\times 10^{6}$ cells). This allows to achieve a harvest of more than $5.3\times 10^{7}$ cells. However, cells can be lost during the process, and the total cell number slips to $5.23\times 10^{7}$ cells, meaning that SBR‐101 is inoculated at a concentration of $8.7\times 10^{4}$ cells/mL.

Between the 2 L Seed Bioreactor (SBR‐101) operating for 144 h and the 10 L Bioreactor (BR‐101) operating for 192 h, no intermediate steps are undertaken, thus no losses are observed. However, to address the issue of nutrient depletion and accumulation of metabolites which can lead to cell death, a switch from batch culture mode to perfusion mode is implemented in the 10 L Bioreactor starting from Day 3. Operating in perfusion mode allows for a constant supply of nutrients while removing toxic by‐products, thereby sustaining cell viability, proliferation and EVs productivity. To enable the perfusion mode, a filter with a 0.45 µm membrane (UF‐101) is placed at the bioreactor exit. This filter facilitates the continuous removal of exhausted medium while retaining the cell‐containing microcarriers, enabling their recirculation back into BR‐101. While cell‐containing microcarriers are too large to cross the 0.45 µm membrane, EVs can pass through. As a result, a rejection coefficient of 0 and 0.5 was assumed for EV1 and EV2, respectively. Consequently, while cells and other unremoved components continue to recirculate back into BR‐101, the removed medium enriched in EVs flows into V‐106, where a selection is made. The flow that enters from days 3 to 6 is immediately discarded (S‐147), while the volume corresponding to the subsequent days is stored and reintroduced later (S‐148). This decision was made for two reasons: (1) EVs should be stored for the shortest duration possible and (2) based on our internal observations, EVs secretion appears to be higher in the later stages of cell growth. Discarding the medium from Days 3 to 6 only results in a 30% loss of the EVs present in S‐146.

For the cells kept in BR‐101, the MSC expansion process assume a 100% cell yield recovery. However, due to factors such as metabolite accumulation, and shear stress caused by the impeller during agitation and the tangential flow filtration perfusion module, cells can die during the culturing process. This impacts cell viability, which needs to be maintained at a high level, preferably above 90% (de Almeida Fuzeta et al. 2020, Lawson et al. 2017, Mizukami and Swiech 2018). Cell dead leads to the release of cellular into the culture medium. In our process simulation, we considered a conservative estimate and assumed that 10% of the cells would lyse, resulting in three types of cellular components, nucleic acids, proteins and cellular debris (mainly lipids and carbohydrates). The stoichiometric coefficients in Equation (6) take into consideration the percentages calculated by Hu (2012).

(6) 100MSC→5DNA/RNA+60Proteins+35Debris

After the extended culturing process, we estimate that a total cell mass of 18.9 g, equivalent to $5.4\times 10^{9}$ cells at a final density of $5.4\times 10^{5}$ cells/mL, will exit the BR‐101 through stream S‐149. This value aligns with cell density values reported in various literature publications (Dos Santos et al. 2014; de Almeida Fuzeta et al. 2020; Adlerz et al. 2019; Lawson et al. 2017; Rafiq et al. 2013). As for MSC‐EVs, 0.11 g were obtained of each EV1 and EV2, resulting in a total of $4.95\times 10^{13}$ particles at a final concentration of $4.95\times 10^{9}$ particles/mL. These values fall within the range of particle concentrations often reported in the literature, which typically range from $10^{8}$ particles/mL to $10^{11}$ particles/mL (Adlerz et al. 2019; Almeria et al. 2022). When considering the EVs reintroduced back into the downstream section for separation/purification, stream S‐165 contains S‐165 $6.92\times 10^{13}$ particles ready to be processed. This naturally increases the particle concentration $6.92\times 10^{9}$ particles/mL, still within acceptable boundaries.

### 2.8 Downstream Processing

The stream exiting the 10 L Bioreactor contains various impurities along with our target products, that must be removed during downstream processing before commercialisation. This section, as mentioned earlier, consists of two separate sectors dedicated to the recovery of MSC or MSC‐EVs, respectively. The first step of the downstream process involves a dead‐end filtration (DE‐103) with a 2 µm pore size membrane. Since MSC‐containing microcarriers are the largest structures present in the conditioned medium, they are retained in the retentate, while MSC‐EVs permeate through the membrane. The DE‐103 equipment separates the downstream process into two distinct sections. The retentate proceeds to the MSC separation/purification section, where cells are detached from the microcarriers and further purified, while the filtrate is directed to the MSC‐EVs separation/purification section. It was assumed that 100% of the MSC‐containing microcarriers remain in the retentate and 87% of the EV1 permeate into the filtrate.

#### 2.8.1 MSC Purification

The retained MSC are transferred into collection tank (V‐102) and undergo three washes with PBS (each lasting 5 min) to remove any remaining undesired components in the stream. Afterwards, a solution of *TrypLE Select CTS* is added to detach MSC from the microcarriers. The microcarriers are then separated from the MSC stream using a 20 µm filtration (DE‐105). The resulting filtrate contains not only MSC but also a range of undesirable components, including metabolites, protease, nucleic acids and others originating from the expansion process, that need to be eliminated before final formulation. To achieve this, the filtrate is concentrated 10 times and diafiltrated with the expansion medium (DF‐103). The recovered MSC, free of unwanted impurities, are then divided into doses. Stream S‐162 contains 16.56 *g* of recovered MSC, corresponding to $4.73\times 10^{9}$ cells. The final formulation involves diluting the MSC in 10 mL of a cryopreservative solution. Assuming a typical MSC dose of more than one million MSC per kg of patient body mass, the final formulation comprises $1\times 10^{8}$ MSC. This means that the process described can potentially generate enough cells to manufacture 47 doses of therapeutic‐grade MSC. However, the viability of cryopreserved MSC products is significantly affected after thawing. The minimum accepted viability for cryopreserved MSC products is 70%, lower than the 90% specified for fresh MSC (Bahsoun, Coopman, and Akam 2019; Hassan et al. 2015). To account for this possible loss, the final doses will contain $1.25\times 10^{8}$ cells, thereby reducing the manufacturing capacity to 37 doses per batch (Figure 2).

#### 2.8.2 MSC‐EVs Purification

The filtrate obtained from the dead‐end filter DE‐103 (S‐150), rich in EVs, also contains residual amounts of cells and cell debris that due to their size or behaviour have managed to pass through the membrane. To ensure the complete removal of these undesirable components, an additional filtration step (DE‐105) using a smaller pore size membrane of 0.8 µm is implemented. EV1, due to their reduced size fully passes the membrane, while some EV2 are lost. In total, there are approximately $4.31\times 10^{13}$ particles circulating in stream S‐163.

After the two consecutive filtration steps (S‐163), the EVs stream still contains several impurities, which include host cell proteins and DNA, which are closely monitored by regulatory agencies. To address this, the stream is further concentrated 10 times and diafiltrated with 7 volumes of 50 mM Tris‐HCl buffer containing 50 mM of L‐arginine, pH 7.5 (DF‐102), using a 500 kDa (0.02 µm) ultrafiltration membrane that retains the EVs (rejection coefficient of 0.98). This DF‐102 step enables the removal of small molecular weight impurities and allows the recovery of approximately 80% of functional EVs. Subsequently, the EVs‐enriched stream undergoes nucleic acid digestion by the addition of an endonuclease and the respective cofactor (V‐105), carried out at room temperature for 2 h. It is assumed that during the nuclease step, 70% of the nucleic acids are digested, and no EVs are lost in the process.

An additional concentration/diafiltration step (DF‐103) using a 500 kDa (0.02 µm) membrane is employed to prepare the EVs stream for anion‐exchange chromatography (AEX) (C‐101) and allows the removal of the endonuclease and small oligonucleotides generated. During this DF‐102 step, the EVs are concentrated 10 times and subsequently diafiltrated with 7 volumes of AEX adsorption buffer, resulting in an overall yield of 80% in functional EVs. By the time we reach the end of the primary purification process (S‐176) a combined yield of 65% is achieved. However, the presence of residual nucleic acids, proteins, and other impurities makes the stream highly heterogeneous, highlighting the critical importance of AEX chromatography to further purify the EVs and eliminate these remaining impurities. Prior to sample loading, the AEX column (Capto Q ImpRes) is pre‐equilibrated with four column volumes (CV) of adsorption buffer (50 mM HEPES buffer, 0.18 M NaCl, pH 7). After sample loading, the column is washed with 7 CV of adsorption buffer and then the product captured by the resin is eluted with 20 CV of elution buffer (50 mM HEPES buffer, 1 M NaCl, pH 7). The EVs are eluted in a volume of 1.5 CV, with a recovery yield of 85%. Using this process, the total EVs purification results in a recovery yield of 55% when compared with S‐163. The final stream, S‐182, contains 0.11 g of purified MSC‐EVs, corresponding to $2.39\times 10^{13}$ particles. As MSC‐EVs are not yet commercialised, there is no consensus among the scientific community regarding the optimal dose for administration. Therefore, an average dosage of $1\times 10^{11}$ EVs per treatment is assumed, meaning that the simulated process can generate enough EVs to manufacture 239 clinical‐grade doses of MSC‐EVs per batch (Figure 2).

## 3 Results and Discussion

Both cell‐based and cell‐free therapies hold significant promise for the future. The global stem cell market, valued at $9.4 billion in 2020, is projected to grow at a compound annual growth rate (CAGR) of 8.8% between 2021 and 2028, reaching $16 billion (Grand View Research I.). Within this, the MSC segment stands out, with an estimated market size of $6.1 billion by 2028, representing a CAGR of 12.6% (Grand View Research I.). This growth underscores the pivotal role of MSC, accounting for over one‐third of the total projected stem cell market value and affirming their significance in future cell‐based therapies. At the same time, EVs‐based therapeutics are gaining attention, with the global EVs market projected to grow at a CAGR of 27.9%, from $174 million in 2020 to an estimated $595 million by 2023 (Mordor Intelligence 2024). The intrinsic link between MSC culture and EVs production creates an opportunity to simultaneously target both markets, leveraging the synergy of these high value bioproducts.

### 3.1 Economic Analysis

For high‐value products such as MSC and MCS‐EVs, achieving commercial success extends beyond demonstrating therapeutic efficacy and associated health benefits. In this competitive market, numerous projects have faced setbacks, even after securing regulatory approval, due to inability to obtain reimbursement from public or private healthcare systems. This challenge is primarily driven by high cost of goods (COG), which translate into prohibitively high selling prices, ultimately limiting market accessibility and adoption (Childs et al. 2020; Driscoll et al. 2017). Therefore, addressing these economic hurdles is as critical as demonstrating therapeutic potential.

### 3.2 Capital Investment

Total capital investment refers to the funds required to initiate, supply, assemble, install, and qualify the manufacturing site, making it ready for operation. Its estimation is highly affected by the type of product to be manufactured. For example, mammalian cell culture, especially for biopharmaceutical production, is more complex and incurs higher costs than microbial cultures. Additionally, the estimation of total capital investment is influenced by the life cycle of the project. In the case of process development, preliminary estimates are usually based on the cost of purchased list and unlisted equipment found in the flowsheet, which was calculated to be 3.553 million Euros using SuperPro Designer. All the equipment used throughout the process is primarily made of stainless steel 316, except for the initial section units, ‘Static Expansion’, where single use technologies were adopted. The latter choice offers a number of advantages, such as reducing contamination risks and lowering capital investment costs, although it increases operating costs.

For the described process, average multiplier values presented by Heinzle et al. were considered, with the exception of the ‘Buildings’ item (Heinzle, Biwer, and Cooney 2007). In that case, the high‐end value was considered due to the requirement of working within cleanrooms, which typically incur high costs associated with such controlled environments. Accordingly, the total plant direct costs (TPDC) were 28.775 million Euros. The total plant cost (TPC) was determined by adding to the TPDC, the engineering (25% TPDC) and construction cost (35% TPDC). The total plant cost amounts to 46.040 million Euros. Adding to this value, a contingency (10% TPC) and a contractor's fee (6% TPC), we obtain a direct fixed capital investment (DFC) of 53.406 million Euros. The total capital investment reaches nearly 65 million Euros and was obtained by considering a working capital of 710 thousand Euros and start‐up and validation costs totalling 10.681 million Euros. This value aligns with the typical investment required for small biotechnology facilities (Harrison et al. 2015).

### 3.3 Operating Costs

The operating costs account for all the ongoing expenses necessary to operate a facility, including raw‐materials, consumables, utilities, factory overheads and other related costs. SuperPro Designer calculates the values directly associated with product manufacturing, which referred to as COG. There is some variability in the literature regarding which expenses are allocated to COG. In this analysis, the annual operating costs, including indirect expenses such as maintenance costs, were divided by the annual production rate to calculate COG per dose. Other expenses, such as Research & Development (R&D), Sales, General & Administrative (SG&A), Royalties and other unspecified costs were not included in this calculation.

The operating costs per batch amount to 531 thousand Euros, with a cycle time of 195.6 h, allowing for 38 batches to be performed per year, resulting in a total annual operating cost of 20 million Euros. Raw materials cost represents 6.42% of the operating costs; labour accounts for 23.95%; facility‐dependent costs are the largest fraction reaching 47.28%; cost for laboratory (offline analysis), quality control (QC) and quality assurance (QA) represent 14.37%; utilities 7.43%; consumables 0.54% and waste treatment 0.01%.

While software appraisal and default values were used for most operations, electricity calculations were based on consumption across all units in all sections. Upstream sections, due to longer operation times, have a higher demand for electricity (Harrison et al. 2015). Indeed, simulation results indicate that approximately 80% of all energy is consumed in the upstream sections, with the ‘dynamic MSC‐EVs and MSC culture’ stage accounting for 76% of that share due to the bioreactors. SuperPro Designer estimations usually exclude overhead lightning, HVAC and cleanroom maintenance costs, which are energy‐intensive spaces necessary for maintaining controlled environments. According to Capparella, pharmaceutical plants have an energy usage intensity of 3819 kWh/m^2^, significantly higher than the 257 kWh/m^2^ needed in a commercial building (Capparella 2013). Assuming a pharmaceutical plant of 2000 m^2^ (including space for laboratory and product manufacture, but excluding commercial areas) we estimate an energy consumption of 2000 MW/h. The price of kW/h was fix at 0.1397€ according on Eurostat's price for non‐household consumers (Eurostat), resulting in an annual expense of more than 1.5 million €.

### 3.4 Profitability Analysis

Increasing process cost‐effectiveness and productivity can be achieved in two ways: (1) through scientific advances that increase the number of collected cells, stimulate the secretion of MSC‐EVs, and reduces the losses observed in downstream processes or (2) by increasing the number of batches per year. Considering the latter approach, the plant is designed to operate throughout the year. The standard process comprises 38 batches, which corresponds to a total of 1406 and 9082 clinical‐grade doses of MSC and MSC‐EVs, respectively.

**FIGURE 4:**
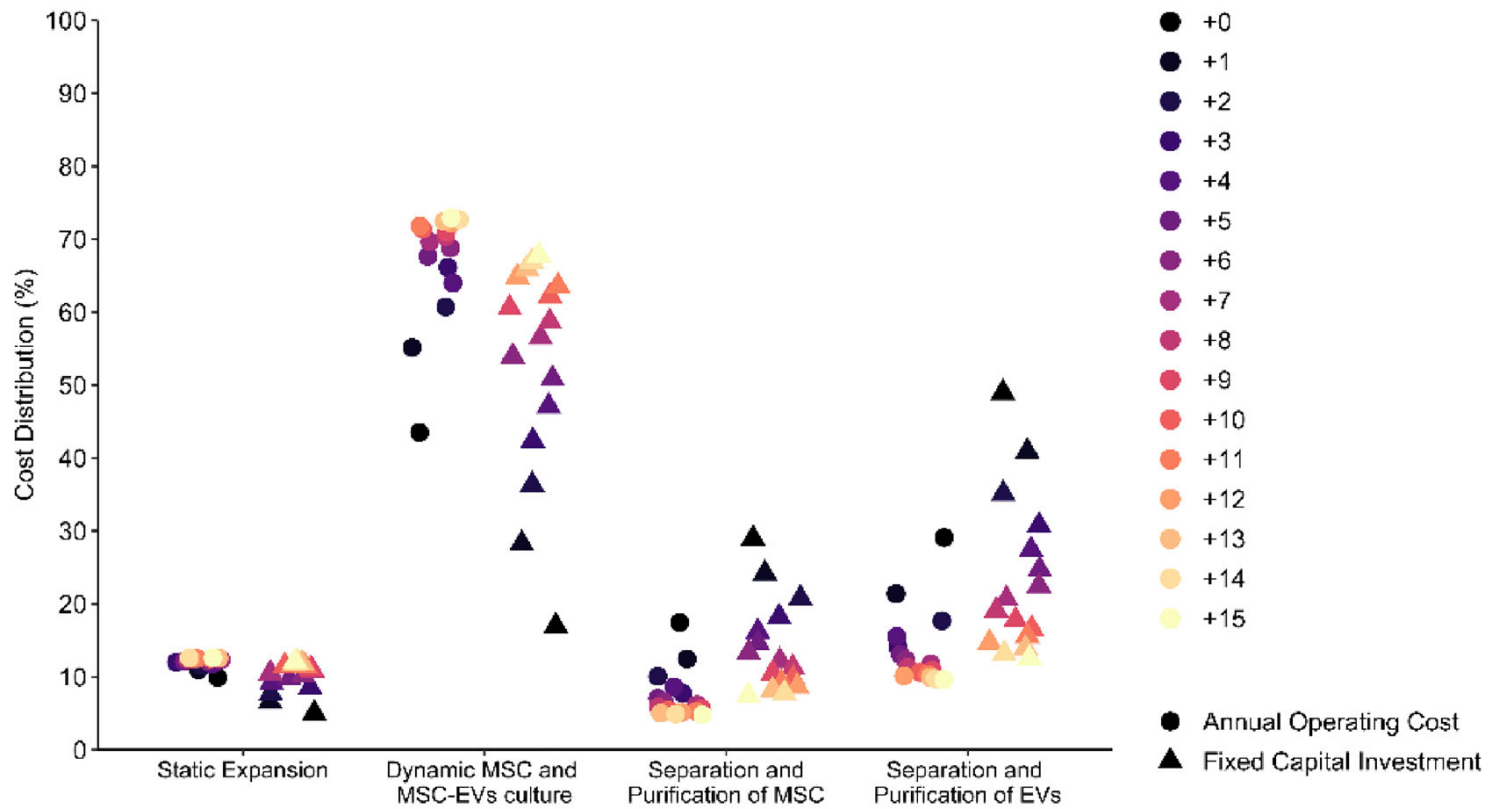
Cost breakdown of fixed capital investment (FCI; ▲) and annual operation cost (AOC; ●) for Process 0 to Process +15. As the number of staggered units increases, the percentage of expenditure tends to shift from the downstream sections—‘Separation and Purification of MSC’ and ‘Separation and Purification of EVs’—to upstream sections, namely ‘Dynamic MSC and MSC‐EVs culture’, absorbing nearly 70% of FCI and AOC by Process +15. MSC, mesenchymal stromal cells; MSC‐derived EVs, extracellular vesicles.

Due to the long culture times, which can extend up to 9 days in the case of 10 L‐Bioreactor, the cultivation stage across all units is by far the limiting step (Figure SA). Cell expansion stages account for 96% of the total batch time, ergo process alternatives started being considered. Running the upstream equipment—T‐175 flasks (TFR‐101), 2 L Seed Bioreactor (SBR‐101), 10 L Bioreactor (BR‐101), 10 L Bioreactor filtration (MF‐101) and 10 L Bioreactor collection tank (V‐101)—in staggered mode reduces waiting times for other equipment, enabling an increase in the number of batches manufactured annually. In the standard process (Process 0), no equipment has been staggered, but in Process +1, one extra unit of each upstream equipment has been considered, and so forth. This strategy is employed until fifteen equipment units have been staggered. In Process 0, over 80% of the capital investment was allocated to downstream processing, which also represents the majority of annual expenses (55%), in line with other biopharmaceutical industries (Hassan et al. 2015). As the number of staggered units increases and the number of batches increases, expenditure tends to shift towards upstream processes (Figure 4) for both capital investment and annual operating costs. This is due to the increased number of additional units and the associated materials and labour expenses, respectively.

**FIGURE 5:**
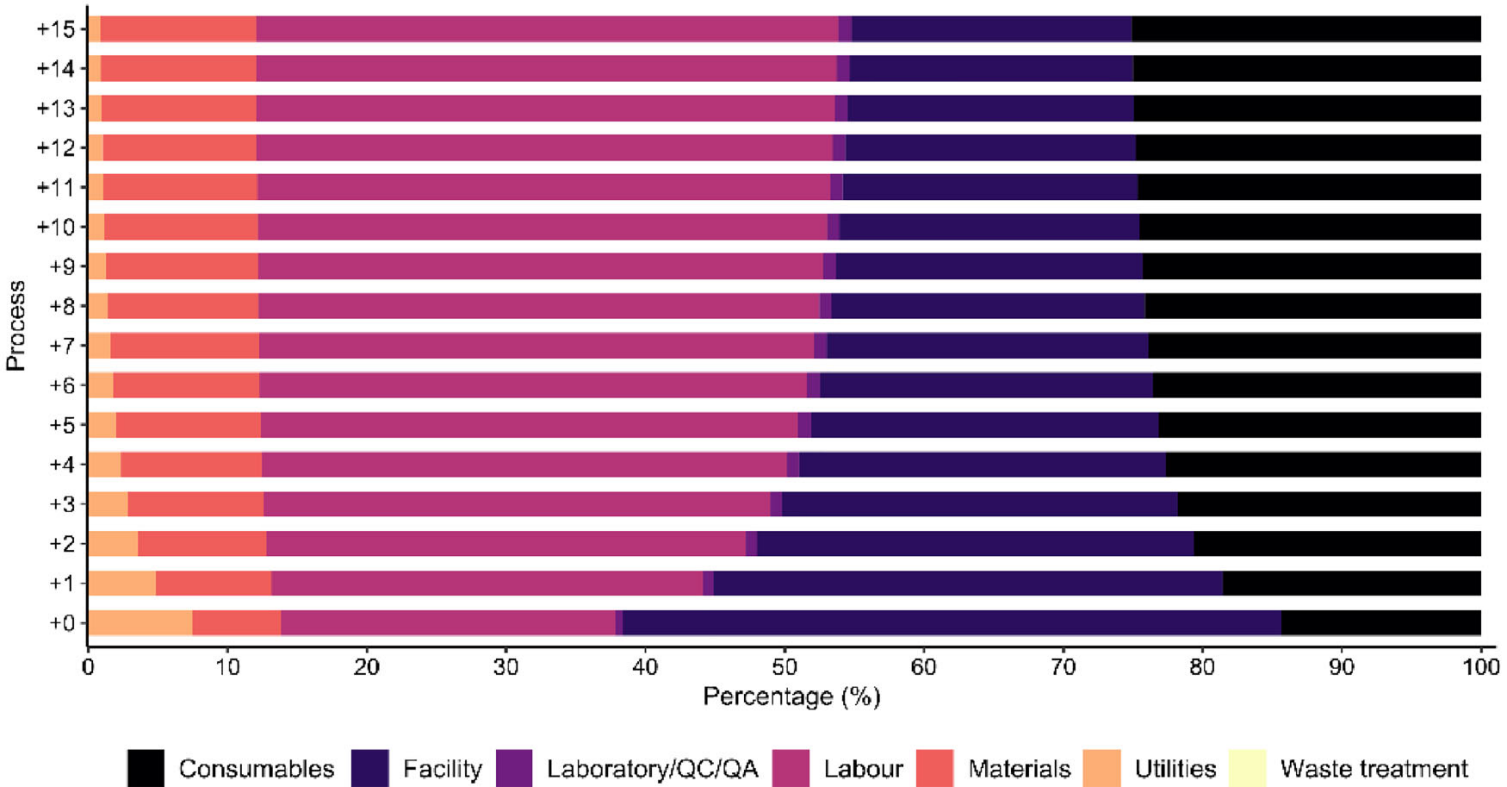
Breakdown of expenses contributing to the annual operating cost (AOC). As the number of staggered units increases, the expenses associated with ‘Laboratory/QC/QA’ and ‘Labour’ surpass those related to the ‘Facility’, becoming the primary sources of expenses.

When examining the breakdown of annual operating costs (AOC), it is observed that facility‐associated expenses, including maintenance, depreciation, insurance, local taxes and facility expenses, account for approximately 50% of the expenses in Process 0. As the number of staggered units increases, the majority of expenses are related to consumables and labour costs (Figure 5).

**FIGURE 6:**
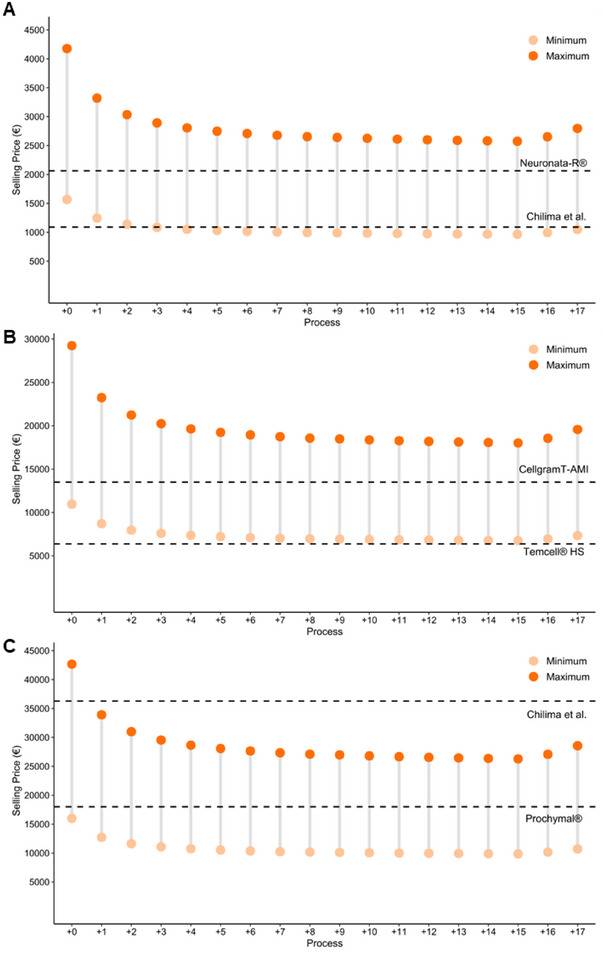
Selling price distribution and comparative analysis of MSC‐based product doses. Selling price distribution of three different doses—10 (A), 70 (B) and 100 (C) millions of MSC‐based products calculated for distinct processes. Process 0 represents the designed process, while the other processes correspond to additional amounts of T‐175 flasks (TFR‐101), 2 L Seed Bioreactor (SBR‐101), 10 L Bioreactor (BR‐101), 10 L Bioreactor filtration (MF‐101) and 10 L Bioreactor collection tank (V‐101) operating in staggered mode. The selling price distribution is calculated considering a COG that ranges from 40% to 15% of sales, representing the minimum and maximum selling prices, respectively. The black dashed lines represent approved cell products or reimbursement decisions for indications that require a similar number of cells, serving as a reference for comparing each MSC dose. MSC, mesenchymal stromal cells.

The strategy of increasing the number of batches manufactured annually increases both capital investment and operating costs, but these costs spread over a higher number of MSC and MSC‐EVs, thus reducing COG (Pereira Chilima, Moncaubeig, and Farid 2018; Jenkins and Farid 2015). The reduction of COG also leads to lower selling prices, which can increase the willingness of healthcare providers to cover therapy costs and improve reimbursement potential. Reimbursement decisions vary between countries and consider factors such as patient clinical improvements and costs to the public health system. For example, in the United Kingdom, the typical reimbursement threshold set by the National Institute of Health and Care Excellence (NICE) ranges from 20,000 to 30,000£ per quality‐adjusted life year QALY), but this value can be extended to higher values, such as 50,000£/QALY (Crabb and Stevens 2016). In Portugal, the threshold ranges from 10,000 to 100,000€/QALY (Perelman et al. n.d.). Figure 6 provides different MSC‐based products and their respective selling prices.

According to previous research, the COG for biological industries, especially for allogeneic cell therapy typically ranges from 15% to 40% of sales revenue. This allows for allocated funds in other company divisions, such as R&D, SG&A and other unspecified expenses, while ensuring plant profitability (Pereira Chilima, Moncaubeig, and Farid 2018; Simaria et al. 2014; Hassan et al. 2015; Smith 2012). Figure 6 displays the spectrum of selling prices for three different MSC dosages: 10 million (Figure 6A), 70 million (Figure 6B) and 100 million (Figure 6C). These prices can be achieved with the proposed manufacturing platform, considering different numbers of staggered units. The selling price pattern follows a decaying function until Process +15, where maximum profitability is achieved, resulting in minimum selling prices of 965, 6755 and 9859€ for the 10, 70 and 100 million dose sizes, respectively. Beyond that point, COG/dose increases, resulting in higher selling price, implying that further process expansion becomes ineffective and unproductive.

The prices listed in Figure 6 for approved MSC therapeutic products serve as benchmarks for comparing the feasibility of the described platform. For the low dose regime of 10 million cells, our proposed selling prices compete with the reimbursement value indicated by Chilima et al., which is approximately $1200 (roughly 1089€) (Pereira Chilima, Moncaubeig, and Farid 2018). For example, Cartistem has a selling price of around 17,100€, four times higher than our maximum selling price in Process 0. When increasing the dose size to 70 million cells, our performance is similar, with prices aligning with those of Temcell HS and Cellgram‐AMI. However, for the former, it requires six staggered units to start competing with the selling price, and even after that, we need to set the selling price at the minimum level to remain competitive. Finally, for the high dose regime of 100 million cells, Prochymal falls entirely within our price spectrum and comfortably below the reimbursement value for myocardial regeneration (Pereira Chilima, Moncaubeig, and Farid 2018).

**FIGURE 7:**
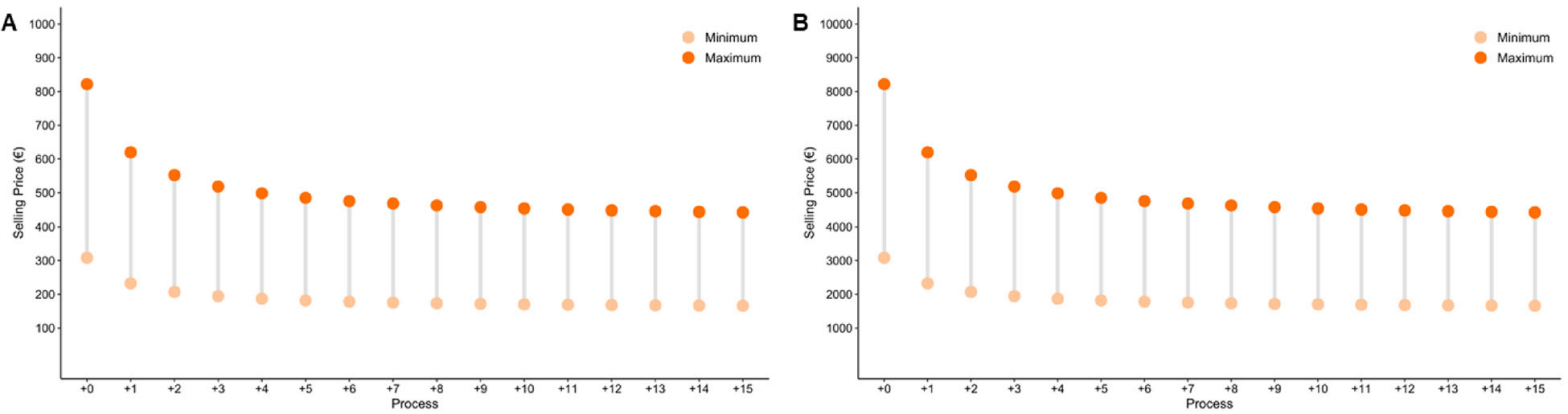
Selling price distribution of EVs‐based product doses in various processes. Selling price distribution for two different doses—$10^{10}$(A) and $10^{11}$ (B) particles—of EV‐based product calculated for distinct processes. Process 0 represents the designed process, while the other processes correspond to additional amounts of TFR‐101, SBR‐101, BR‐101, UF‐101 and V‐101 now reads T‐175 flasks (TFR‐101), 2 L Seed Bioreactor (SBR‐101), 10 L Bioreactor (BR‐101), 10 L Bioreactor filtration (MF‐101) and 10 L Bioreactor collection tank (V‐101) operating in stagger mode. The distribution is based on a COG equal to 40% of sales (minimum selling price) and 15% of sales (maximum selling price). EVs, extracellular vesicles.

For MSC‐EVs products, the situation differs significantly as no therapy has currently received approval from governmental agencies, making it impossible to establish a benchmark using existing products. However, the decay behaviour of the COG/dose reported for MSC is again observed here. In our analysis, two dose sizes of $10^{10}$ and $10^{11}$ particles/vial are considered (Figure 7). Due to the higher production ratio of particles/cell, the COG/dose is significantly more reasonable, meaning that if MSC and MSC‐EVs products compete for the same target condition, industries may redirect their production line towards EVs, as it gets more cost‐effective and more attractive to seek reimbursement approval.

**FIGURE 8:**
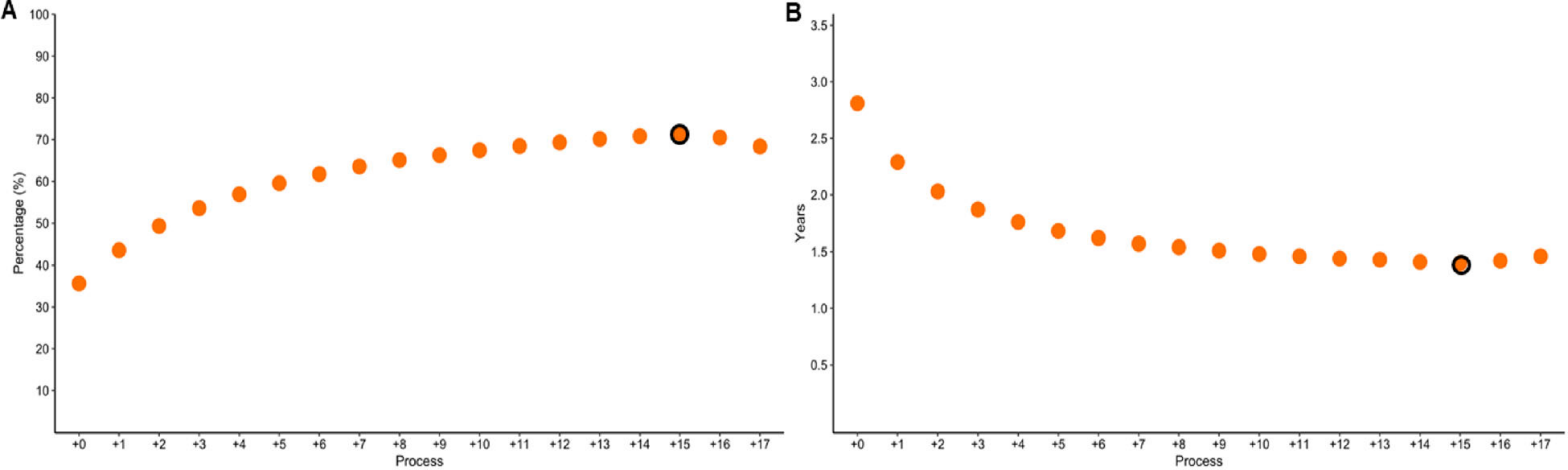
Economic analysis of different processes. (A) Return on Investment (ROI) variation across different processes. Process +15, highlighted in black, achieves the highest ROI at 71%. (B) Payback time variation across different processes. As in A, Process +15 also highlighted, minimizes the Payback time to 1.4 years.

In the current simulation, we have designed a platform focused on the co‐production of MSC and MSC‐EVs therapeutic‐grade products. Both are considered revenue sources and can be sold in the range calculated in Figures 6 and 7. Taking the most conservative scenario, where both products are sold at the minimum price, meaning the company absorbs 40% of COG and hence reduces profitability, the payback time for Process 0 is 3 years, which decreases to less than 1.5 year by Process +15 (Figure 8). Although promising, this value will inevitably increase as investments in R&D, SG&A and other non‐discriminated expenses are taken into account.

## 4 Final Remarks

In recent years, there has been a notable increase in the number of clinical trials involving MSC with a few MSC‐based products already reaching the market. More recently, the therapeutic potential of MSC derivatives, particularly their ‘secretome’ rich in EVs, has gained significant attention. Clinical trials data indicate that significant quantities of these products will be required for widespread clinical use, justifying the need of scalable MSC‐EVs manufacturing platforms. To leverage synergies within upstream processing, that requires MSC production for MSC‐EVs isolation, MSC themselves were considered as a co‐product. This strategy reduces waste and maximises resource utilisation. Given the less complex downstream processing requirements for MSC compared to MSC‐EVs, most of the costs are incurred in the purification of EVs. However, the co‐production approach allows for cost‐sharing between the two products, contributing to lower overall COGs and enabling more competitive selling prices.

The results demonstrate that the proposed manufacturing platform can effectively deliver MSC‐based products with different dose sizes—10, 70 and 100 million cells—at minimum selling prices ranging from 965 to 4177€ for smaller doses, and from 9859 to 42,673€ for larger doses. These prices position the products as competitive alternatives to established cell‐based therapies currently available in the market. For MSC‐EVs, the calculated selling prices are substantially lower than those of MSC, ranging from 1659 to 3082€ for doses containing $10^{11}$ particles and from 166 to 309€ for smaller doses with $10^{10}$ particles. This highlights economic advantage of MSC‐EVs‐based approaches in comparison to MSC therapies.

Despite the promising economic results, there remains room for improvement. For example, expanding the utilisation of single‐use technologies throughout the entire upstream process, rather than limiting them to the static expansion stage, could further reduce fixed capital investment. Additionally, optimising downstream processes to improve efficiency and reduce costs could enhance economic viability. The designed manufacturing process demonstrates strong potential as a competitive alternative to established production and purification platforms, enabling the generation of clinical‐grade doses that can rival existing cell‐based therapies. However, it is important to consider that certain products may require additional handling and treatment, depending on the target condition which could influence cost and reimbursement dynamics. It is important to acknowledge that final selling prices and reimbursement decisions are shaped by detailed cost‐effectiveness studies, which evaluate therapeutic benefits relative to patient outcomes. These factors contribute to the observed price variability among products with similar dose regimes. Overall, the findings of this study provide robust evidence feasibility and competitiveness of the proposed co‐production platform. Future refinements, such as the adoption of advanced technologies and further optimisation of process parameters, have the potential to enhance economic performance, improve scalability and solidify the market positioning of the platform.

## Author Contributions

**Ricardo M. Silva**: conceptualization (equal), formal analysis (lead), investigation (lead), writing–original draft (lead). **Sara Sousa Rosa**: validation (equal), writing–review and editing (equal). **José Santos**: supervision (equal), validation (equal), writing–review and editing (equal). **Ana M. Azevedo**: conceptualization (equal), funding acquisition (equal), supervision (equal), validation (equal), writing–review and editing (lead). **Ana Fernandes‐Platzgummer**: conceptualization (equal), funding acquisition (lead), project administration (lead), supervision (equal), validation (equal), writing–review and editing (equal).

## Conflicts of Interest

The authors declare no conflicts of interest.

## Supporting information



Supporting Information

## Data Availability

The data that support the findings of this study are available from the corresponding author upon reasonable request.
