# Supplementary material for: Enabling Mesenchymal Stromal Cells and Their Extracellular Vesicles Clinical Availability—A Technological and Economical Evaluation
Source: J Extracell Biol. 2025 Mar 17;4(3):e70037. doi: 10.1002/jex2.70037 (PMC11913891; doi:10.1002/jex2.70037)
Supplement: Supplementary file 1 — Supporting Information [file JEX2-4-e70037-s001.docx]

**Enabling mesenchymal stromal cells and their extracellular vesicles clinical availability – a technological and economical evaluation**

Ricardo M. Silva, Sara Sousa Rosa, José A. L. Santos, Ana M. Azevedo and Ana Fernandes-Platzgummer

# **Supplementary Information**


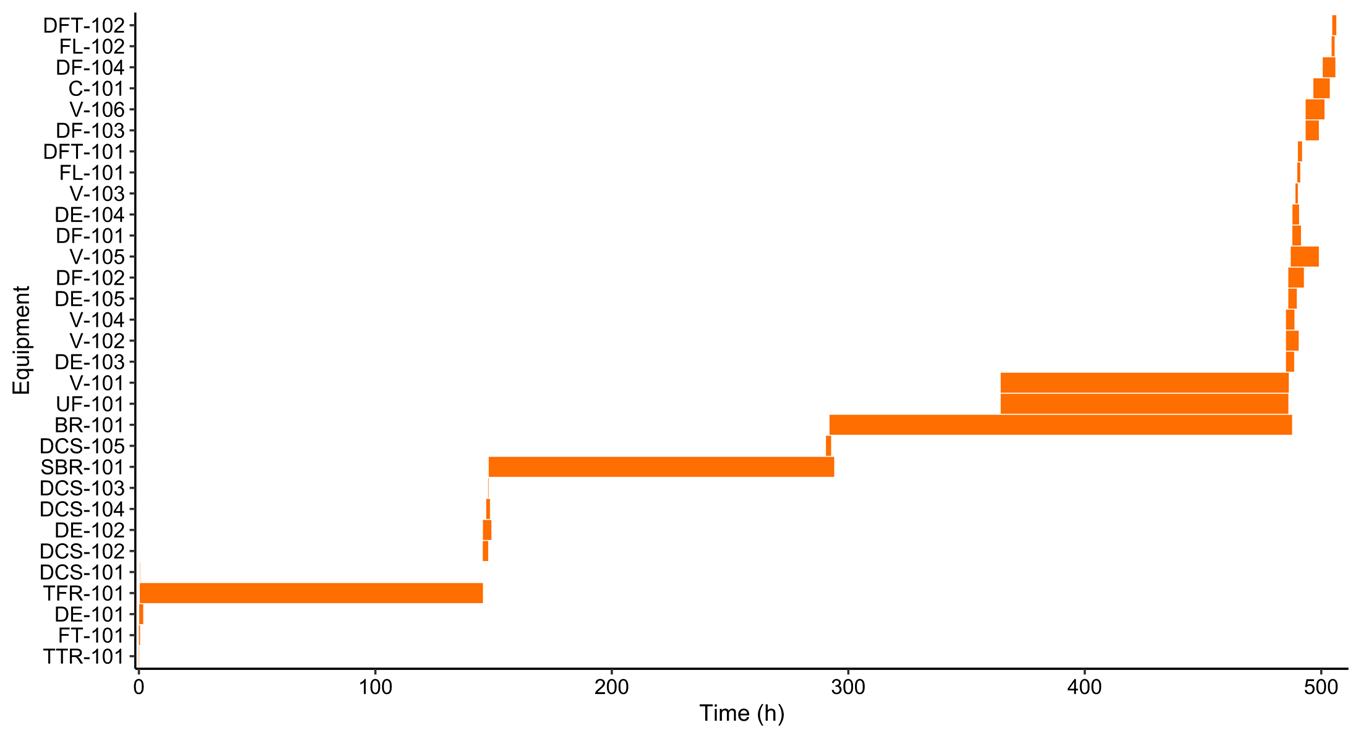


**Figure A. 1:** **Equipment occupancy time for the duration of a batch.** The batch has a duration of 506.37h. TTR-101: Test Tube; FT-101: Thaw module; DE-101: Dead-end filtration; TFR-101: T-flasks units; DCS-101: Storage units; DE-102: Dead-end filtration; DCS-102: Storage unit; DCS-103: Storage unit; DCS-104: Storage unit; DCS-104: Storage unit; SBR-101: Seed Bioreactor 2L; DCS-105: Storage unit; BR-101: Bioreactor 10L; UF-101: Filtration; V-101: Storage unit; DE-103: Dead-end filtration; V-102:Storage unit; V-104: Storage unit; DE-105: Dead-end filtration; DF-102: Diafiltration; V-105: Storage unit; DF-101: Diafiltration; DE-104: Dead-end filtration; V-103: Storage unit; FL-101: Filling MSC; DFT-101: Discrete freezer MSC; DF-103: Diafiltration; V-106: Storage unit; C-101: Chromatography; DF-104: Diafiltration; FL-102: Filling EV; DFT-102: Discrete freezer EV.
